# On the Tongue as a Means of Diagnosis

**Published:** 1855-01

**Authors:** 


*On the Tongue as a means of Diagnosis.* By Thos. Newman, Esq., M. R. C. S. E., L. S. A.

Every practitioner is in the habit of looking at the tongue in all cases of internal, and in most cases of external disease. From a glance at its general appearance, he forms an idea of the amount of irritation which may exist in the digestive apparatus, large or small intestines. In a surgical case, also, the tongue informs what may be the amount of constitutional irritation present. I believe, however, that the tongue is an organ which requires looking into much more minutely than is commonly done, that certain portions of it may be allotted to particular diseases, and that our remedies may consequently be given with greater precision. In making this assertion, I am advancing no new theory, but merely supporting an old one, as well as a neglected one, proposed by Dr. Ridge. I read his work, tested it to the best of my ability, and have arrived at the following conclusions:

1. The tongue points out to us the particular organ affected in all chylopoietic derangements.
2. It indicates the seat of disease in the respiratory apparatus.
3. The tongue gives unmistakable appearances in affections of the circulation and media thereof.
4. It is a most valuable guide in fevers.

Many of the readers of the Lancet may not have seen the book I allude to, and therefore, without some little explanation, will scarcely understand some of the terms used in this communication. Dr. Ridge divides the tongue longitudinally into three parts: central laterals, small portions on either side the raphé; laterals, to the outer side of the foregoing; and edges. He then divides these, by means of transverse lines, into—

Anterior,
Second,
Central,
Posterior, } fourths.

giving a part of the anterior fourth of the central laterals to the "tip," and the junction of the anterior and second fourth, to the "oval."

The central laterals show the respiratory organs.

The laterals, the chylopoietic viscera.

Edges, the brain.

The tip shows the state of the large intestines.

The oval is given to the pleura.

The heart seems to influence the whole of the tongue.

In support of my first conclusion, I would wish to call attention to any ordinary case of dyspepsia, with sluggish liver. Here the tongue may almost always be found covered with a thin, white flim, the whole length of the laterals, these portions being thrown prominently forward, and most clearly defined; and in nearly all those cases where sickness and pyrosis have been present, the posterior and central fourths have been most thickly coated. It sometimes occurs in this affection that the tongue is slightly coated over its whole surface; but on desiring the patient to protrude it forcibly, the posterior fourth of the laterals is seen thickly furred. As the affection extends to the liver and duodenum, the laterals become coated the whole length; and on the application of remedies, embracing the stomach and liver, the disease quickly yields. If the posterior and central fourths are alone affected, our treatment must be confined to the stomach alone, and, I believe, with a certainty of success. In one instance of stricture of the œsophagus, near the cardiac orifice of the stomach, I observed the posterior fourth of the laterals to be thickly coated, and as the disease advanced, doubtless affecting the extremity of the stomach, the furring progressed anteriorly. Again: in a case of malignant disease of the pylorus, which rapidly extended to the duodenum, and formed a mass of disease easily felt externally, the laterals were the only parts of the tongue coated, the other portions of the organ being perfectly clean and glassy. I could produce many other instances of derangement of the primæ viæ which my note-book informs me invariably presented the same appearances, but I will not occupy the pages of the Lancet with "twice-told tales."

Secondly. The appearance of the tongue in cases of phthisis (which have come under my observation) has been varied, not in the position, but in the degree of coating. We are to remember that the central laterals are the portions of the tongue given to all thoracic disorders, as well as to those of the larynx and trachea, and it is to these parts we must look for our indications of disease. Accordingly, we shall find them covered with fur thrown up, as it were, by injection of the vessels, and clearly defined. I am not now referring to pneumonia and pleuritis, (neither of which diseases I have seen during the last two years,) but to cases of tubercular deposit, and ultimate softening, with all its attendant symptoms. With regard to the *degree* of coating, I have found, that although well marked as to position, it has been in some of the recent cases less than in more advanced ones. I will quote a case, illustrating in a remarkable manner that the diseases of the air-passages, as well as of the substance of the lungs, are indicated by the state of the central laterals.

"A patient of broken-down constitution was afflicted with secondary symptoms—ulceration of the fauces and soft palate among the number. After the usual remedies had been applied, and persisted in for some time, the parts became sound, and the case progressed favorably, when suddenly great difficulty of breathing came on, approaching to strangulation, and rendering it a matter of doubt whether the operation of tracheotomy should not be performed. The symptoms were relieved by anti-spasmodics and sedatives in powerful doses. The attacks recurred at intervals of fourteen hours during three days, and was subdued by the same treatment. On the sixth day, the disease was apparently checked, but only for a short time, for within a few days unmistakable symptoms of gangrene of the lungs made their appearance, and rapidly carried off the patient. I watched this case narrowly, and I found that immediately the difficulty of breathing came on then did the posterior and middle fourths of the central laterals become coated, which coating disappeared on the cessation of the urgent symptoms; but after the lungs had been attacked, a thick creamy deposit covered the whole of the central laterals as far forward as the tip."

Thirdly. In all cases of chlorosis, where the system is deprived of proper nutrition, the tongue assumed a flabby aspect; it is tremulous, and its papillæ, particularly those at the tip and sides of the tongue, assume a fringe-like appearance, but are pale, and almost œdematous. In these cases there is a loud aortic bruit, with the bruit de diable. Upon the application of the proper remedies in such cases, the papillæ first became very slightly colored, then contract into their normal dimensions, and, lastly, the whole tongue assumes a healthier aspect some time before the struggle of the patient returns.

In some instances of hypertrophy, with and without dilatation, the tongue becomes covered with sulci, more particularly on either side of the raphé. It is also dryer than usual, thickened, and appearing as if it labored under some congestive disease. I have had no opportunity of seeing a case of aneurism lately, but I have frequently searched for and found disease of the heart after observing these deep fissures of the tongue.

Fourthly. A large number of cases of fever occurred in this city last autumn. It was always a simple type at the onset, presenting all the usual symptoms of derangement of the primæ viæ, with rapid pulse, hot skin, &c. After the initiative measure of purging and an emetic, I found the tongue remaining, if possible, more coated than before; and in some cases repeated the purge and emetic given, at the same time, saline aperients and diaphoretics. After four or five days the coating of the tongue became dry, but did not disappear, and I was compelled to have recourse to stimulants, which performed their task most satisfactorily, for the tongue became moist, and in twenty-four hours, was nearly clean. In all my future cases I pursue this course. I gave, first, an emetic, then a purge, and waited until the second day; I then examined the tongue, and if I found the tip (which is the part given to the large intestines) in a clean state, in ever so small an extent, I gave ammonia and bark, and had the pleasure of seeing my patients recover more rapidly than I ever remember under any other plan of treatment.

In these cases I ought perhaps to have waited until the tongue gave evidence of the small intestines having recovered themselves. I did not do so, and never lost a case afterwards.

It is not only in fevers that we must study the appearance of the tip of the tongue. It is a most valuable guide in constipation of the bowels. It is then slightly furred, and covered with papillæ, which are so injected as to appear some distance above the fur. In these cases violent purges only render matters worse, and it is only by gentle and long-continued medicines that we shall restore our patient.

In affections of the small intestines we all are accustomed to the appearance of the tongue; but on looking at the anterior fourth of the laterals, by its injection or non-injection, by its ulceration or non-ulceration, shall we detect the true state of the mucous membrane of the small intestines as far as the cœcum.

In conclusion, I beg to say that with respect to the brain my observations have been so limited that I have not ventured to deduct any inferences, but that whatever I have now advanced I have repeatedly proved. I have not given any anatomical proofs of the intimate connection of the several divisions of the tongue with the parts they represent, as I consider that would be trespassing on Dr. Ridge's province, and I trust that my professional brethren will derive as much pleasure and profit from his book as I have done; or, rather, that it may encourage abler heads than mine to investigate his *theories* for themselves; and, in the end, to impart them to the profession as FACTS, as both he and I believe them to be.—*Southern Medical Journal*, from *London Lancet.*

